# Flying between Sky Islands: The Effect of Naturally Fragmented Habitat on Butterfly Population Structure

**DOI:** 10.1371/journal.pone.0071573

**Published:** 2013-08-01

**Authors:** Sandhya Sekar, Praveen Karanth

**Affiliations:** Centre for Ecological Sciences, Indian Institute of Science, Bangalore, India; Assignment tests; Tuscia University, Italy

## Abstract

High elevation montane areas are called “sky islands” when they occur as a series of high mountains separated by lowland valleys. Different climatic conditions at high elevations makes sky islands a specialized type of habitat, rendering them naturally fragmented compared to more continuous habitat at lower elevations. Species in sky islands face unsuitable climate in the intervening valleys when moving from one montane area to another. The high elevation shola-grassland mosaic in the Western Ghats of southern India form one such sky island complex. The fragmented patches make this area ideal to study the effect of the spatial orientation of suitable habitat patches on population genetic structure of species found in these areas. Past studies have suggested that sky islands tend to have genetically structured populations, possibly due to reduced gene flow between montane areas. To test this hypothesis, we adopted the comparative approach. Using Amplified Fragment Length Polymorphisms, we compared population genetic structures of two closely related, similar sized butterfly species: *Heteropsis oculus*, a high elevation shola-grassland specialist restricted to the southern Western Ghats, and *Mycalesis patnia*, found more continuously distributed in lower elevations. In all analyses, as per expectation the sky island specialist *H. oculus* exhibited a greater degree of population genetic structure than *M. patnia*, implying a difference in geneflow. This difference in geneflow in turn appears to be due to the natural fragmentation of the sky island complexes. Detailed analysis of a subset of *H. oculus* samples from one sky island complex (the Anamalais) showed a surprising genetic break. A possible reason for this break could be unsuitable conditions of higher temperature and lower rainfall in the intervening valley region. Thus, sky island species are not only restricted by lack of habitat continuity between montane areas, but also by the nature of the intervening habitat.

## Introduction

“Sky islands” are a sequence of high elevation mountain areas separated by lowland valleys [1]. The high elevation areas are characterized by different climate, and hence different vegetation. Sky islands can be regarded as continental equivalents of oceanic islands, with mountain tops acting as cradles of evolution, and the intervening valleys with very different climate, akin to a sea of alien vegetation [1]. Species that inhabit the high elevation montane areas have been shown to be physiologically incapable of dispersing through low lying valleys [2].

There are many examples of restricted gene flow between sky islands from the well studied Madrean archipelago of sky islands. One of the most extreme examples is that of genetic diversification over a small spatial scale in the jumping spider *Habronattus pugillis* [3]. Reduced gene flow has also been reported in other taxa, like the longhorn cactus beetle [4], the New Mexico ridge-nosed rattlesnake, *Crotalus willardi obscurus* [5] and the black bear *Ursus americanus* [6]. Similar results have been obtained in other sky island systems like the Rocky mountains (for instance, reduced gene flow in the alpine plant *Sedum lanceolatum* [7] and the montane grasshopper *Melanoplus oregonensis* [8]).

The high elevation areas of the Western Ghats mountain range in southern India form one such sky island group. The Western Ghats (WG) is a 1600 km long mountain chain, which extends from north to south along the west coast of peninsular India. The mountain chain is broken by only one large gap: the 40 km long Palghat Gap. The southern Western Ghats are on an average much higher than the northern Western Ghats, with Anaimudi in south Kerala being the highest peak, with an elevation of 2295 m. These high elevation montane regions have an altitude of over 1900 m and are characterized by a special type of vegetation - the shola-grassland complex. The sholas are stunted forests with an upper story of trees, a twisted second story and a dense shrub layer of saplings; they occupy ridges between mountaintops. The shola forests are surrounded by undulating grasslands, consisting of fire and frost-resistant species of grasses [9].

The sky islands of the Western Ghats exhibit a high degree of endemism, with fifty endemic plant genera, half of which are monotypic, and with almost 50% of India’s 206 amphibian species endemic to this region [10]. This makes it more crucial to study the gene flow patterns among populations of species isolated in such a naturally fragmented system. The phylogeographic structure of the white bellied shortwing (*Brachypteryx major*), a bird endemic to the shola forests of the Western Ghats, showed maximal genetic differentiation across the widest valley in the Western Ghats - the 40 km wide Palghat Gap [11]. Some montane plant species have also been shown to exhibit genetic differentiation among sky island populations [12], [13].

These studies suggest that species confined to sky islands tend to have structured populations, ostensibly due to reduced gene flow across the sky islands. However, most of these studies have looked at a single sky island species, and more importantly, have not compared it with non-sky island species. Comparing species which inhabit the naturally disjunct sky island habitat, with similar species that inhabit the more continuously distributed low and mid elevation areas, can offer some insights into whether sky island species are capable of maintaining connectivity between different populations.

Butterflies in the Western Ghats make an interesting system to address such questions, because the composition of the butterfly community changes drastically between the mid-elevation and montane areas. Genetic structure as a result of habitat specialization has been shown in butterflies earlier, especially in the case of montane species, like in the high altitude meadow butterflies *Parnassius smintheus* [14], and *Erebia melampus* [15]. However, these studies have not compared sky island species with continuously distributed species from lower elevations.

In this study, we looked at the population genetic structure of two closely related butterfly species from peninsular India, both from the subtribe Mycalesina (Family Nymphalidae, subfamily Satyrinae). *Heteropsis oculus* is an endemic species found only to the south of the Palghat Gap in the Western Ghats, and restricted to montane areas above 1200 m. The closely related *Mycalesis patnia* is a generalist woodland species that occupies lower and mid elevation areas. The two species are similar with respect to all traits that affect dispersal in butterflies, except for the kind of habitats they occupy [16]: both species are of approximately the same size, their larvae are generic grass feeders, they have multiple generations throughout the year, and are on the wing for most of the year. Both species are weak fliers, and do not undertaking noticeable long distance flights [17]. The relatedness and high similarity between the two species makes this a robust comparison; thus a difference in genetic structure between them can be attributed to the difference in the spatial orientation of their habitat with greater confidence.

In this study, we predict that the sky island specialist *Heteropsis oculus* will have more geographically structured populations, because of the spatial orientation of montane forests (disjunct habitat) along the Western Ghats. *Mycalesis patnia*, being more continuously distributed in the lower elevations, will have a more homogenous population structure. Such a result will provide greater support to the hypothesis that sky island species tend to have structured populations due to reduced gene flow across suitable habitat patches. We sampled both species along the Western Ghats, and also sampled *Heteropsis oculus* intensively across one mountain range, the Anamalais, to enumerate gene flow between sky island populations separated by short distances.

## Materials and Methods

### Study Species

Both *M. patnia* and *H. oculus* belong to subtribe Mycalesina, which is part of subfamily Satyrinae and Family Nymphalidae. The subfamily Satyrinae in peninsular India has about 20 species in the low and mid elevation areas, which are almost completely replaced by just two species in the higher elevation areas [17]. Even if other species do occur, their densities are very low. The sub tribe Mycalesina has about six species in low and mid elevation areas, which are almost completely replaced above 1200 m by *Heteropsis oculus* (henceforth HO) to the south of the Palghat Gap, and *Heteropsis adolphei* to the north of the Palghat Gap. Both *Heteropsis* species are endemic to the sky islands to the north and south of the Palghat Gap respectively [18]. *Mycalesis patnia* (henceforth MP) is a generalist woodland species endemic to the Western Ghats and Sri Lanka that occupies evergreen, deciduous and scrub forests in the lower and mid elevation areas. HO belonged to the genus *Mycalesis* until recently, when a molecular phylogenetic study placed it in *Heteropsis*, another genus in the same subtribe [19].

### Sampling

**Figure 1:**
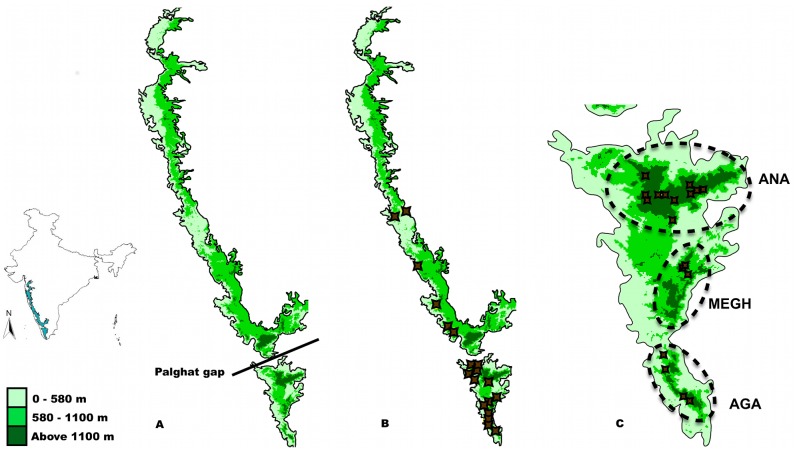
Maps showing sampling locations. Inset: A map of India showing the position of the Western Ghats. Panel A is an outline of the Western Ghats, showing the altitudinal gradient. A scale shows the altitudes represented by the different shades of green. The largest break in the mountain chain, the 40 km long Palghat Gap, is also shown. Panel B shows sampling locations for *Mycalesis patnia.* Panel C is the outline of the Western Ghats south of the Palghat Gap, showing sampling locations for *Heteropsis oculus.* The three shola complexes sampled in this study are indicated with dashed ellipses. (ANA – Anamalai complex; MEGH – Meghamalai complex; and AGA – Agastyamalai complex).

Both study species were collected from across their range in the Western Ghats: MP from northern Karnataka to south Kerala, and HO from the south of the Palghat Gap in Kerala and Tamil Nadu. We conducted active searches and also set up traps using fermented bait made from a mixture of over-ripe bananas, sugarcane juice and rum. For intensive sampling of HO, three hill complexes that are high enough for shola forests south of the Palghat Gap were demarcated, using an elevation map of the Western Ghats. These included the Anamalai-Palni complex (ANA), the Meghamalai-High Wavies complex (MEGH) and the Agastyamalai complex (AGA) (Figure 1 panel C). The ANA complex was sampled most intensively for HO, resulting in 58 individuals over a distance of about 60 km. The full HO dataset had 99 individuals sampled over a straight-line distance of about 200 km.

MP was sampled from the low elevation areas corresponding to the above shola complexes (see Figure 1 panel B). A total of 57 samples, including 45 from the south of the Palghat Gap, and 12 from the north, were collected. In all areas, we took care to sample from many different patches to capture maximal genetic diversity within the area. All captured individuals were killed immediately and stored in paper envelopes as voucher specimens. We would like to thank the forest departments of Kerala, Karnataka and Tamil Nadu for collection permits. For a full list of collected specimens, and the GPS locations of where they were collected, refer to Table S1 in the Supporting Information.

### Lab Techniques

We first tried out sequencing of mitochondrial DNA markers (cytochrome oxidase b – cyt b, cytochrome *c* oxidase subunit I - COI, and control region). All these markers did not show any intraspecific variation within the specimens we had from the Western Ghats. So, we decided to use the Amplified Fragment Length Polymorphisms (AFLPs) technique instead. The AFLP protocol is known to be sensitive to contamination, especially from microbial DNA, which is abundant in the digestive system of the insect [20]. Hence, care was taken to use only two legs for the extractions. The extraction protocol was adapted from Reineke *et al* [21]. All extracts were quantified using a spectrophotometer NanoDrop ND-1000 (NanoDrop Technologies, Wilmington, DE, USA), and then diluted to 20 ng/µl to ensure uniform amounts of DNA in the starting material.

The restriction and ligation reactions were accomplished in a single step. The total DNA was digested with 1 unit of MseI (New England Biolabs) and 5 units of EcoRI (New England Biolabs), along with the adaptors. The MseI adaptors were 5′-GAC GAT GAG TCC TGA G-3′ (forward) and 5′-TAC TCA GGA CTC AT-3′ (reverse). The EcoRI adaptors were 5′-CTC GTA GAC TGC GTA CC-3′ (forward) and 5′-AAT TGG TAC GCA GTC TAC-3′ (reverse). The restriction-ligation reaction was carried out at 37 degrees for 4 hours. The products were diluted with 90 µl Tris-EDTA before the next step. The pre-selective PCR used the EcoRI primer E/A (5′-GACTGCGTACCAA TTCA-3′) and the MseI primer M/C (5′GATGAGTCCTGAGTAAC-3′), with the PCR amplification parameters as follows: 72°C for 2 min, 30 cycles of denaturing at 94°C for 30 sec, annealing at 56°C for 30 sec, extension at 72°C for 2 min, and final elongation at 60°C for 10 min.

The product of the pre-selective PCR was run on a 2% agarose gel to check for amplification, indicated by a smear in the 100 to 1000 bp range. Successful samples were diluted with 100 µl using Tris-EDTA, and used in the selective amplification. AmpliTaq Gold (Applied Biosystems) was used during the selective PCR to minimize amplification error. The PCR parameters were: 94°C for 2 min, and 36 cycles of denaturing at 94°C for 30 sec, annealing for 30 sec and extension at 72°C for 2 min. Annealing was initiated at 65°C and then reduced by 0.7°C per cycle for the next 12 cycles, and maintained at 56°C for the next 23 cycles.

Four selective primer pairs were chosen, after testing over 20 combinations of primer pairs. Selective primers with both 2 and 3 extra nucleotides than the preselective primers (+2 and +3 primers [22]) were tested. The nucleotides to be added were chosen based on the relative numbers of A and C [23]. The EcoRI primers were labeled with fluorescent dyes. The final four primer pairs for each species are given in Table 1. All oligonucleotides were procured from Eurofins Genomics India Private Limited. All PCR reactions were carried out in a BioRad thermocycler.

**Table 1:** List of selective primers used in the AFLP analysis of MP and HO.

Mycalesis patnia			Heteropsis oculus		
Labeled	Unlabeled	Number of loci	Labeled	Unlabeled	Numberof loci
E4	M1	126	E4	M1	136
E4	M7	152	E4	M3	113
E7	M1	121	E5	M7	140
E7	M7	139	E7	M7	127
Total loci: 538			Total loci: 516		

E4 (FAM): GACTGCGTACCAATTC-CAA; E5 (HEX): GACTGCGTACCAATTC-CAT; E7 (FAM): GACTGCGTACCAATTC-CT. Here, fluorescent dye used for labeling is mentioned in brackets. FAM: 6-FAM Fluorescein (blue dye) and HEX: hexachlorofluorescein (green dye). M1: GATGAGTCCTGAGTAAACAT; M3: GATGAGTCCTGAGTAAA CTA; M7: GATGAGTCCTGAGTAAA-AT.

After the selective PCR, the samples were diluted by 1∶15, and run on the 16 capillary Applied Biosystems 3130xl sequencer at the National Centre for Biological Sciences, Bangalore. 0.6 µl of the sample was mixed with 0.5 µl of GeneScan-500 ROX size standard (Applied Biosystems) and 12 µl of Formamide. The plate was centrifuged to mix the contents, and then the DNA denatured by heating at 95°C for 3 min, followed by a quick chill on ice. The Applied Biosystems Standard Dye Set D was used to detect peaks. The electropherograms were visualized, and edited where necessary, using Peak Scanner (Applied Biosystems). The sizing table, containing information on the intensity and size of the peaks, was obtained from Peak Scanner, and imported into RawGeno [24] for binning and scoring.

In order to minimize error, the same person (SS) did all the lab work, from extraction to loading the samples into the sequencer. All the samples could not be worked on at the same time, because the field work was done in batches. To ensure that between run variability did not affect results, positive and negative controls were run and error rates checked at the start of each batch of runs. When needed, all the electropherograms from one primer pair were normalized before analyses using the R package AFLPScore [25]. About 10% of the total number of samples (both species combined) were used as blind replicates to estimate error rates (using AFLPScore), which came to about 4%. The estimated error rate of 4% is considered acceptable for publication [26].

### Analyses

**Figure 2:**
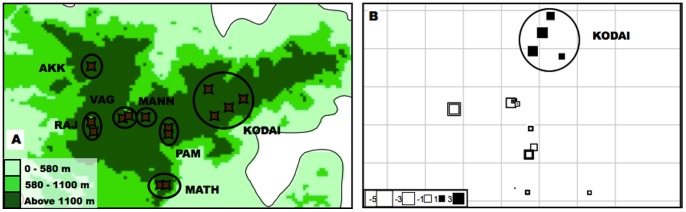
sPCA plots for the Anamalai population of *Heteropsis oculus.* Panel A shows the populations created for the population based analyses. Panel B is a representation of the first axis sPCA scores. The graph denotes the scores using squares; colour of the squares denotes positive (black) or negative (white) spatial autocorrelation, and the size of the squares denotes the magnitude of genetic variance. The squares are overlaid on a connection graph that represents the sampling design. If there was genetic differentiation between populations, squares of similar colour will clump together in one population. The individuals from Kodaikanal (denoted with a circle, named KODAI) are well differentiated from the other individuals in the Anamalais.

During the analysis, we compared two AFLP datasets: the MP dataset, with all the individuals of *M.patnia* (*n*  = 57), and the HO dataset, with all the individuals of *H.oculus* (*n*  = 99). Additionally, since we sampled the Anamalais region intensively for HO, we performed all the analyses for a reduced dataset of HO, which included only individuals collected from the Anamalais (the HO-ANA dataset, *n* = 58; see Figure 2 Panel A for a map showing sampling locations). For all datasets, we carried out 1) individual based analyses to obtain overall patterns in the dataset - genetic diversity, spatial autocorrelation, isolation by distance and clustering; and 2) population based analyses after assigning individuals into populations - genetic differentiation and assignment tests.

### Overall Patterns

#### a) Spatial autocorrelation

The spatial autocorrelation statistic *r*, which is closely related to Moran’s *I*, was estimated using the methodology from [25], implemented in GenAlex [26]. Like Moran’s *I*, *r* also assumes values ranging from –1 to +1. Eight uniform distance classes of 100 km each was used for the MP dataset, five distance classes of 50 km each for the HO dataset, and six distance classes of 10 km each for the HO-ANA dataset. The significance of *r* in each distance class was established using 1000 permutations [27]. The result of the permutation tests was analyzed using both one-tailed and two-tailed tests. The differences between the two datasets were estimated using three statistical tests, following the methodology described in Banks (2012) [27].

#### b) Isolation by distance

Associations between geographic and genetic distance in both species were tested using a Mantel’s test, implemented in the package ade4 version 1.4 [28] in the statistical language R v2.14 [29]. Jaccard’s distance measure was used to compute a genetic distance matrix, which is the best at dealing with homoplasy in binary data [30]. A simple Euclidean distance matrix was calculated for geographic distance. Both distance matrices were calculated using the R package “proxy” v 0.6 [31]. The Mantel test was run for 1000 permutations to test for significance.

#### c) Clustering analyses

Since Bayesian clustering algorithms like STRUCTURE make a lot of assumptions (Hardy-Weinberg equilibrium, linkage equilibrium and regular sampling) while performing the clustering of genotypes [32], multivariate clustering approaches were adopted. A recently developed protocol, the spatial principal component analyses (sPCA) technique, was used [33]. sPCA uses a synthetic variable derived from variation in allele frequencies and spatial information. Spatial information is usually provided in the form of a connection network, chosen based on the sampling protocol. Since sampling was clumped for MP and HO datasets, the inverse distances connection network was used; for HO-ANA, since the sampling was more evenly placed, a Gabriel graph connection network was used [33].

Two permutation tests were used to test for positive and negative spatial autocorrelation: the Gtest (test for global structures, positive spatial autocorrelation), and Ltest (test for local structures, negative spatial autocorrelation). The PCA was performed in the package ade4 version 1.4 [28], and sPCA in the package adegenet version 1.3 [34], both implemented in the statistical language R v2.14 [29].

### Population Based Analyses

**Figure 3:**
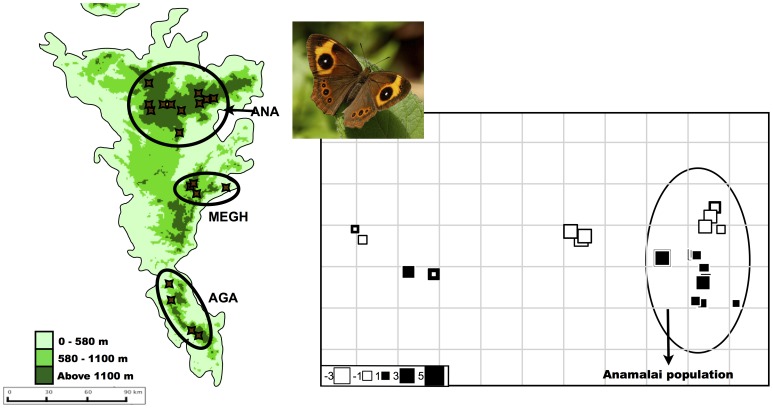
sPCA plots for *Heteropsis oculus.* Panel A shows the populations created for the population based analyses. Panel B is a representation of the first axis sPCA scores. The graph denotes the scores using squares; colour of the squares denotes positive (black) or negative (white) spatial autocorrelation, and the size of the squares denotes the magnitude of genetic variance. The squares are overlaid on a connection graph that represents the sampling design. If there was genetic differentiation between populations, squares of similar colour will clump together in one population. There is some clumping in the Anamalai population (shown in a circle), which is further explored in the next figure. Inset: photograph of *H. oculus.* Credit: Balakrishnan Valappil.

In order to carry out the population-based analyses, we grouped samples from each dataset into populations by putting individuals from the same area together. For deciding the cutoff for placing individuals to a group, we used the first quartile from a distribution of geographic distances between sampling coordinates. This gave rise to three groups in the HO dataset (see panel A of Figure 3): Anamalai group (ANA, *n* = 58), Agastyamalai (AGA, *n* = 21) and Meghamalai (MEGH, *n* = 20). The HO-ANA dataset clustered into seven groups (see panel A of Figure 2): Pambadam shola (PAM, *n* = 9), Mannavan shola (MANN, *n* = 7), Akkamalai (AKK, *n* = 4), Rajmala (RAJ, *n* = 6), Vaguvaraiyar shola (VAG, *n* = 10), Mathikettan shola (MATH, *n* = 11) and Kodaikanal (KODI, *n* = 11). The MP dataset had five groups (see panel A of Figure 3): samples from Anamalais hill complex and the surrounding plains (ANA, *n* = 15), samples from Meghamalais and Periyar (MEGH, *n* = 15), samples from Agastyamalais (AGA, *n* = 15), samples from northern Karnataka (NKAR, *n* = 7) and samples from Wayanad and Coorg (WAC, *n* = 5).

#### a) Genetic diversity

Genetic diversity parameters were estimated using the program AFLPSurv [35]. Allele frequencies were estimated using a Bayesian method with non-uniform prior distribution of allele frequencies [36], under the assumption of Hardy-Weinberg genotypic proportions. This method has been demonstrated to be the best available for dominant markers as it has the least bias [37]. Heterozygosities were further calculated using a method specifically for dominant markers [38].

#### b) Genetic differentiation

The genetic differentiation between the groups in each dataset was estimated using both band-based and allele-frequency based *F_ST_* [37], [39]. AFLPSurv was used to estimate allele frequencies, as described earlier, and the allele frequency values were used in the calculation of *F_ST_*. Pairwise genetic distances (Nei’s D, after [38]) were also estimated. The significance of the *F_ST_* values was evaluated using a permutation test, with 1000 permutations. The program Arlequin [40] was used to perform the Analysis of Molecular Variance (AMOVA), in order to calculate the molecular variance found among groups, within groups, within populations and within individuals.

#### c) Assignment tests

Assignment tests were carried out in AFLPOP [41]. A relatively high minimum log-likelihood difference (MLD) of 1 was used, to reduce chances of misassignment [42]. The value for ε was chosen as 0.001, as per the default settings. When individuals were allocated to a population other than the one where they were sampled from, the AFLPOP simulation option was used to assess the probability of incorrect assignment, using 1000 random specimens. The simulation gives a *P* value of allocation to the second population; when this *P* value is low (<0.01), the chance of correct allocation to the second population is high. In some cases, the software cannot place an individual into any of the populations with high confidence; in such cases, the individual is considered an immigrant from outside the sampled metapopulations.

## Results

### Overall Patterns

#### a) Spatial autocorrelation

The MP dataset did not show significant spatial autocorrelation in the first distance class (MP first distance class, 0–100 km, *r* = 0.004, *P* = NS; HO first distance class, 0–50 km, *r* = 0.003, *P* = 0.003) while the HO dataset did, though of very low magnitude. The third distance class (100–150 km) was significant in the HO dataset, but the *r* was low (0.008). However, the spatial autocorrelation correlograms of MP and HO did not differ significantly (*r* MP = 0.004, 95% bootstrap CI = −0.005 to 0.006, *r* HO = 0.003, 95% bootstrap CI = −0.002 to 0.003; T2 (first distance class) = 0.253, *P* = NS; Omega = 11.08, *P* = NS). In the HO-ANA dataset, the first four distance classes do not show significant spatial autocorrelation. However, the fifth and sixth distance classes (above 40 km) showed significant negative spatial autocorrelation. The complete results of the spatial autocorrelation analysis are given in Table S2.

#### b) Isolation by distance

Both the MP and HO datasets exhibited significant IBD; however, the correlation value for HO was more than twice that of MP (MP Mantel *r* = 0.1168, *P* = 0.014; HO Mantel *r* = 0.272, *P* = 0.0001). The graphs for the Mantel test are presented in Figure S1. IBD is significant in HO-ANA (Mantel *r* = 0.218, *P* = 0.0005), with an elevated *r* value at a Euclidean distance of 0.3 − 0.4.

#### c) Clustering analysis

**Figure 4:**
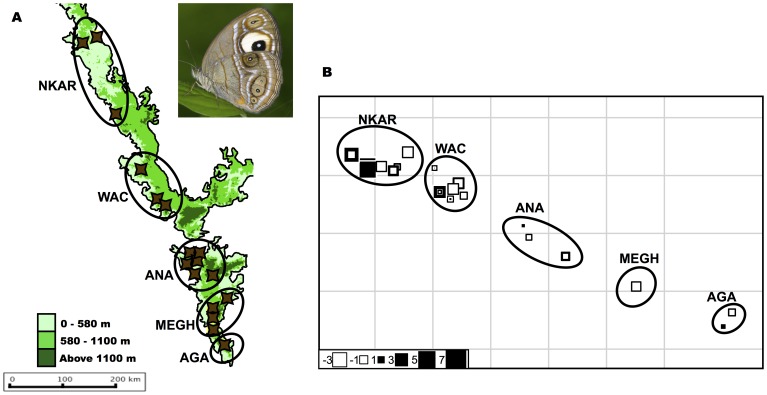
sPCA plots for *Mycalesis patnia.* Panel A shows the populations created for the population based analyses. Panel B is a representation of the first axis sPCA scores. The graph denotes the scores using squares; colour of the squares denotes positive (black) or negative (white) spatial autocorrelation, and the size of the squares denotes the magnitude of genetic variance. The squares are overlaid on a connection graph that represents the sampling design. If there was genetic differentiation between populations, squares of similar colour will clump together in one population. This is clearly not the case for *M. patnia.* Inset: photograph of *M. patnia.* Credit: Krushnamegh Kunte.

Based on an examination of the screeplot of sPCA values of the MP dataset (see Figure S2, part A), only the first sPCA eigenvalue (variance = 4.302, Moran’s *I* = 0.655) was retained. There were no significant clusters retrieved in this analysis (see Figure 4), and both global and local tests were not significant (Gtest max(t) = 0.034, *P* = NS; Ltest max(t) = 0.044, *P* = NS).

In the case of the HO dataset, only the first sPCA eigenvalue was retained (variance = 3.211, Moran’s *I* = 0.868) (see Figure S2, part B for the screeplot). The Gtest was significant while the Ltest was not (Gtest max(t) = 0.0205, *P* = 0.001; Ltest max(t) = 0.017, *P* = 0.189). However, the significant global structure did not translate into any geographic structuring (see Figure 3). Close examination of the Anamalais group (encircled in Figure 3), shows the presence of some pattern, which was further explored in the HO-ANA dataset.

For HO-ANA, the first sPCA eigenvalue (variance = 3.158, Moran’s *I* = 0.887) was retained, since it was far higher than all the others (see Figure S2, part C for the screeplot). Global structures were significant, but not local structures (Gtest max(t) = 0.034, *P* = 0.029; Ltest max(t) = 0.030, *P* = 0.498). In Figure 2, the samples represented by black squares inside the circle (which encompasses sampling locations from the Kodaikanal subpopulation) are different from the other samples, which are all lighter colored; this indicates a progressive genetic differentiation between Munnar and Kodaikanal.

### Population Based Analyses

#### a) Genetic diversity

The genetic diversity parameters for all the datasets are summarized in Table 2. The percentage polymorphic loci (PLP) was similar across all the populations of the MP dataset, ranging from 47.8% in WAC to 57.9% in NKAR. The expected heterozygosity (*H_e_*) was similarly uniform, ranging from 0.15 to 0.17; the total heterozygosity was 0.162. In HO, the PLP was about 42% in the ANA and MEGH populations, but as high as 51.6% in the AGA population. The total heterozygosity was 0.115, with *H_e_*ranging from 0.105 to 0.12. There was higher variation in genetic diversity among sub populations in the HO-ANA dataset: the PLP varied from 38.5% in AKK, to about 71% in MATH. The total heterozygosity was 0.167, with *H_e_*varying from 0.14 to 0.19.

**Table 2:** Genetic diversity in the MP, HO and HO-ANA datasets.

Dataset	Population	PLP	*H_e_* (± SD)
MP	AGA	52.2	0.167 (0.005)
	ANA	49.6	0.154 (0.005)
	MEGH	56.6	0.170 (0.005)
	NKAR	57.9	0.166 (0.005)
	WAC	47.8	0.155 (0.005)
HO	AGA	51.6	0.112 (0.004)
	ANA	41.8	0.121 (0.004)
	MEGH	42	0.105 (0.004)
HO-ANA	AKK	38.5	0.157 (0.007)
	KODI	65.6	0.147 (0.006)
	MANN	54.9	0.156 (0.006)
	MATH	70.9	0.194 (0.006)
	PAM	66	0.177 (0.006)
	RAJ	46.7	0.158 (0.007)
	VAG	66	0.179 (0.006)

Here, PLP is the percentage of polymorphic loci, and *H_e_* is the expected heterozygosity (± standard deviation). Population names are abbreviated as follows: AGA – Agastyamalais, ANA – Anamalais, MEGH – Meghamalais, NKAR – North Karnataka, WAC – Wayanad/Coorg complex, AKK – Akkamalai, KODI – Kodaikanal, MANN – Mannavan shola, MATH –Mathikettan shola, PAM – Pambadam shola, RAJ – Rajmala, VAG – Vaguvaraiyar.

#### b) Genetic differentiation

Genetic differentiation among populations in MP dataset was not significant in the allele-based method (see Table 3a) and only marginally significant in the band-based method (see Table S3 a). Nei’s genetic distance was less than 0.001 among all populations (see Table 3a). The HO-dataset exhibited low, but significant differentiation among its three constituent populations, with a low overall allele-based *F_ST_* of 0.0217, P<0.0001 (see Table 3b) and an overall band-based *F_ST_* of 0.043, P<0.0001 (see Table S3 b). Nei’s D values were also very low, around 0.002 (see Table 3b)).

**Table 3:** Genetic differentiation matrices among populations in a) MP b) HO and c) HO-ANA datasets.

a) MP dataset												
Populations		AGA		ANA			MEGH		NKAR		WAYCOO	
AGA		–		0.0016			0.0011		0.0019		0.0008	
ANA		0.0085		–			0		0		0	
MEGH		0.0056		0			–		0		0	
NKAR		0.0096		0			0		–		0	
WAYCOO		0.0043		0			0		0		–	
Overall *F_ST_* = 0.0015, *P = *0.064												
**b) HO dataset**												
**Populations**			**AGA**			**ANA**				**MEGH**		
AGA			–			0.0024				0.002		
ANA			0.0183			–				0.004		
MEGH			0.0162			0.0304				–		
Overall *F_ST_* = 0.0217, *P*<0.0001												
**c) HO-ANA dataset**												
**Populations**	**AKK**		**KODI**		**MANN**	**MATH**		**PAM**		**RAJ**		**VAG**
AKK	–		0.0044		0	0		0		0		0
KODI	0.0239		–		0.0018	0.0071		0.0023		0.0045		0.0067
MANN	0		0.0098		–	0		0		0		0
MATH	0		0.0349		0.0074	–		0		0		0
PAM	0		0.0123		0	0		–		0		0
RAJ	0		0.0246		0	0		0		–		0
VAG	0		0.0341		0	0		0		0		–
Overall *F_ST_* = 0.002, *P*<0.0001												

Nei’s D genetic distance is presented in the upper triangle, and allele-based *F_ST_* values in the lower triangle.

In HO-ANA, the overall *F_ST_* value was 0.002, at P<0.0001; see Table 3c for more details. The *F_ST_* between KODI and the other sub populations is the major contributor to the overall *F_ST_* value. *F_ST_* is 0.034 between KODI and MATH, and KODI and VAG, while the *F_ST_* among other sub-populations is 0. Similar patterns can be seen with Nei’s D (Table 3c)) and band-based *F_ST_* (see Table S3 c). The only other pair of sub-populations showing differentiation are MATH and MANN, though not as high as the values between KODI and other sub-populations.

#### c) Assignment tests

In the MP dataset, 15 out of the 57 individuals could not be assigned to any population, and 15 were assigned to the population from which they were sampled. Simulations were performed for the 27 individuals that were not assigned to the populations from which they were sampled. 22 out of these 27 individuals were assigned with high probability to the population they were sampled from, making the total number of individuals correctly assigned, 37 (65%). The remaining five individuals were assigned with high probability to populations they were not sampled from: two from ANA were assigned to MEGH, two from WAC to MEGH and one from MEGH to NKAR.

Of the 99 individuals in the HO dataset, 16 could not be assigned to any one population with confidence. 70 samples were assigned to the population from which they were sampled. However, simulations showed that the remaining 13 individuals had the highest probability of being assigned to the population they were sampled from.

In the HO-ANA subset, 9 out of the 58 individuals could not be assigned to one population with confidence, and only 15 of the remaining 49 individuals were correctly assigned: 9 to KODI, and 6 to MATH. But, simulations showed that all the individuals except one were assigned with high probability to the population they were sampled from. Only one individual from AKK was assigned to MATH with high confidence.

## Discussion

In this study, we compare the population genetic structure of two butterfly species that occupy habitats with different levels of continuity. Among these species, HO is a sky-island specialist distributed in naturally disjunct habitat, and MP is distributed in relatively more continuous lowland forests. The two species are similar in all other traits that affect dispersal ability; thus a difference in genetic structure can be attributed to a difference in habitat continuity. We first established that the overall genetic diversity in both species was not significantly different. The two AFLP datasets can thus be compared meaningfully. Importantly, the *H_e_* values from this study are comparable to the values obtained in other studies that have used AFLP [43]–[45].

### The Effect of Spatial Orientation of Suitable Habitat Patches

Spatial autocorrelation was not observed in MP, while HO exhibits significant spatial autocorrelation, even though the correlation value is very low. The clustering analysis sPCA did not retrieve clusters in either MP or HO. However, the sPCA analysis of HO showed a positive global structure (significant G test), indicating significant positive spatial autocorrelation, as opposed to the lack of global structures in MP. Genetic differentiation was not significant in MP, and significant, though low, between the three populations of HO. The same pattern was observed in the assignment tests, with the algorithm unable to confidently assign individuals to the correct populations in MP, because of a homogenous genetic structure. There were, however, a high percentage of correct assignments in HO, showing more restricted dispersal. Taken together, these results indicate that HO, the sky island species, showed a higher population genetic structure than MP, the mid and low elevation species. A summary of the analyses is given in Table 4.

**Table 4:** Comparison of population genetic analyses performed for MP and HO datasets.

Analysis	Support for hypothesis
Spatial autocorrelation	Not significant in MP, significant in HO
IBD	Mantel's *r* 2.3 times greater in HO
sPCA	Gtest not significant in MP, significant in HO
Band-based *F_ST_*	Not significant in MP, significant in HO
Allele-based *F_ST_*	Not significant in MP, significant in HO
AMOVA	Variation between populations explained two times more genetic variation in HO
	2.4 times higher percentage of correct assignment in HO

The difference between the genetic structure of MP and HO can be attributed to the spatial orientation of the habitats they occupy, because the two species are similar in all other respects. HO, the sky island specialist inhabiting a naturally disjunct habitat exhibits more geographic structuring than MP, which inhabits the more continuously distributed low and mid elevation forests in the Western Ghats. Thus our results support the hypothesis that sky islands tend to have structured populations due to reduced gene flow across the sky islands.

Similar results of reduced gene flow among sky islands have been observed in the montane butterflies, *Erebia melampus* [15] and *Erebia Euryale* [46] in Europe. A case in point is *Parnassius smintheus* from the Canadian rockies [14], where the patches of suitable high altitude meadows have been pushed further apart by habitat fragmentation, resulting in a drop in gene flow. The landscape level changes in the Western Ghats need to be investigated further, but further reduction in connectivity between sky islands is a likely scenario.

As mentioned above, MP seems to posses a uniform genetic structure across the Western Ghats. More intense sampling may show different patterns; but, MP has been sampled across most of its range in the Western Ghats, and the genetic diversity values are comparable to global values. This indicates that sampling has not been a problem. Such a lack of genetic structure has been observed in other butterfly species, especially among Satyrines: examples are *Chazara briseis* [47] and *Melanargia galathea* [48].

Also, some butterfly species can have an innate ability to disperse over long distances, depending on the spatial distribution of resources necessary for their survival: sites where males and females can find each other and mate, for oviposition where larval food plants are proximately distributed, and where adults can find food and survive to be able to perform both these activities [49]. This has been demonstrated in *Parnassius apollo* [50]. Some species can travel through patches of unfavorable habitat even if they prefer a particular habitat type. A study on butterflies of the subfamily Ithomiinae (Family Nymphalidae) in the cloud forest fragments in the Columbian Andes showed that butterflies moved through the coffee plantations around forests, flying faster through sunny rather than shady patches, but still being able to move through unsuitable habitats [51].

### Genetic Differentiation among Sky Islands in HO

Within HO, an interesting pattern was revealed. In the HO-ANA dataset, KODI samples formed a distinct cluster in the PCA plot when the first and third axes were used. Also, KODI samples formed a distinct cluster in the sPCA analyses, indicating differentiation between KODI and all other sub-populations (which are all situated in Munnar, see Figure 2). The uniqueness of the Kodaikanal population within the Anamalai subset of HO is also evident by the other analysis parameters. Spatial autocorrelation and isolation by distance within the Anamalais samples are evident at about 40 km, the distance of KODI from other populations. Genetic differentiation (both *F_ST_* measures and Nei’s D) in the HO-ANA dataset is low, but the overall values are contributed by the differentiation between KODI and the other sub-populations. Even in assignment tests, only one individual was assigned to a population from which it was not sampled.

The presence of such genetic structuring over a short distance of about 40–50 km is further evidence of the reduced connectivity between sky islands, which is limited in comparison to connectivity between lower elevations. In the white-bellied shortwing, a bird species also found in the sky islands of the Western Ghats, a study of song structure [52] demonstrated that birdsong was beginning to diverge between Kodaikanal and the nearby Grass Hills. The samples of *H.oculus* from Akkamalai (which is adjacent to Grass Hills) are closely related to the individuals from Munnar, and well differentiated from the Kodaikanal samples. It is possible that similar forces are at play here. Anthopogenic habitat fragmentation is probably causing songs to diverge because populations are getting isolated (though with no corresponding genetic differentiation yet) in the white-bellied shortwing, and genetic differentiation in *H.oculus*.

The reasons for a break in gene flow between Kodaikanal and Munnar can only be speculated upon. The following information was obtained from the high resolution vegetation map [53] and from the bioclimatic maps [54] of the Western Ghats, both developed by the French Institute. The altitude of the areas sampled from Munnar (average 1730 m) and Kodaikanal (average 1845 m) are similar, though Kodaikanal is slightly higher. Temperatures are thus similar, with the mean temperature in the coldest month being less than 13°C in both places. The geology is also similar, with the bedrock composed of metamorphic rocks from the Precambrian, with gneiss and granite; soils are “ferralitic (laterites) to fersialitic (red soils), with a massive development of kaolinite as a product of rock weathering where the annual soil water balance is consistently positive (i.e. above 1,200 mm rainfall)” [53]. The vegetation is also similar, with patches of montane shola-grasslands, interspersed with plantations of tea, eucalyptus, wattle and pine.

The main difference is the amount and seasonality of rainfall in both places, which manifests as a strong west-east gradient in the number of dry months, from Munnar to Kodaikanal. Munnar experiences the Allepey-Mangalore rainfall regime, characterized by heavy monsoon showers in June, July and August, with 2000 to 5000 mm rainfall annually, (mean of 3594 mm), spread over 144 rainy days, and an average dry season length of 3 months per year. As one moves east to Kodaikanal, there is a succession of different rainfall regimes: 1) a strip of the Shimoga regime (1200 to 1500 mm annual rainfall, with 3–6 dry months, less than 100 rainy days and temperature of coldest month between 13.5°C and 16°C), and 2) the Madurai-Pollachi regime (mean annual rainfall 875 mm, with 4–8 dry months, about 66 rainy days and temperature of coldest month between 16°C and 23°C), until Kodaikanal, which is a special case of the Madurai-Pollachi regime due to increased rainfall over a few days (mean annual rainfall 1571 mm, with 0–4 dry months and about 92 rainy days, and temperature again about 13.5°C). This probably means that HO, being adapted to the high rainfall and low temperature in Kodaikanal, is unable to disperse to Munnar due to the intervening dry area.

This brings to light an important fact: apart from the spatial orientation of suitable habitat patches, the nature of the intervening habitat is equally important in determining whether dispersal (and thus gene flow) can occur in a particular species. Replicating studies of this kind with other taxa across the Munnar – Kodaikanal gradient, and in other areas of the Western Ghats that show such a strong gradient, like the Nilgiris, will help further our understanding of habitat connectivity between sky islands. This assumes further importance given the high degree of endemism in the sky islands of the Western Ghats, especially in the case of plants. While there may be a difference between species based on species biology, comparative studies such as these lay the ground for making generalizations regarding the ability of a species to maintain genetically viable populations.

There is now information regarding two species that inhabit the sky islands of the Western Ghats: an endemic bird, and an endemic butterfly. Both studies show that gene flow is restricted at these high elevations. The high elevation areas of the Western Ghats have been systematically converted into plantations from British times: tea plantations in Munnar started in the 1870s, and the replacement of grasslands with timber trees (pine, wattle and eucalyptus) in Kodaikanal started in the early 1900s. The montane shola forests and grasslands have been swamped with invasive exotic species that have changed the composition of native vegetation [55], [56]. Human occupation at high densities (about 261 people per square km) has resulted in extensive modification of both the montane landscape, and the intervening ‘valley’ areas. There is a strong need to conserve unmodified areas in the Western Ghats as a whole, and the unique shola grassland mosaic in particular; for this endeavor, establishing the connectedness between sky islands across taxa is important.

## Supporting Information

Figure S1 **Mantel test for a) HO-FULL and b) HO-ANA showing variation in** ***r*** **in different distance classes.**
(PDF)

Figure S2 **Eigenvalues and screeplots for the sPCA analyses.**
(PDF)

Table S1 **Sampling locations for both species.**
(PDF)

Table S2 **Full results of the spatial autocorrelation analysis.**
(PDF)

Table S3 **Band-based** ***F_ST_*** **values between populations in all datasets.**
(PDF)
