# Supplementary material for: Flying between Sky Islands: The Effect of Naturally Fragmented Habitat on Butterfly Population Structure
Source: PLoS One. 2013 Aug 1;8(8):e71573. doi: 10.1371/journal.pone.0071573 (PMC3731288; doi:10.1371/journal.pone.0071573)

**Figure S1.** Mantel test for A) HO-FULL and B) HO-ANA showing variation in *r* in different distance classes

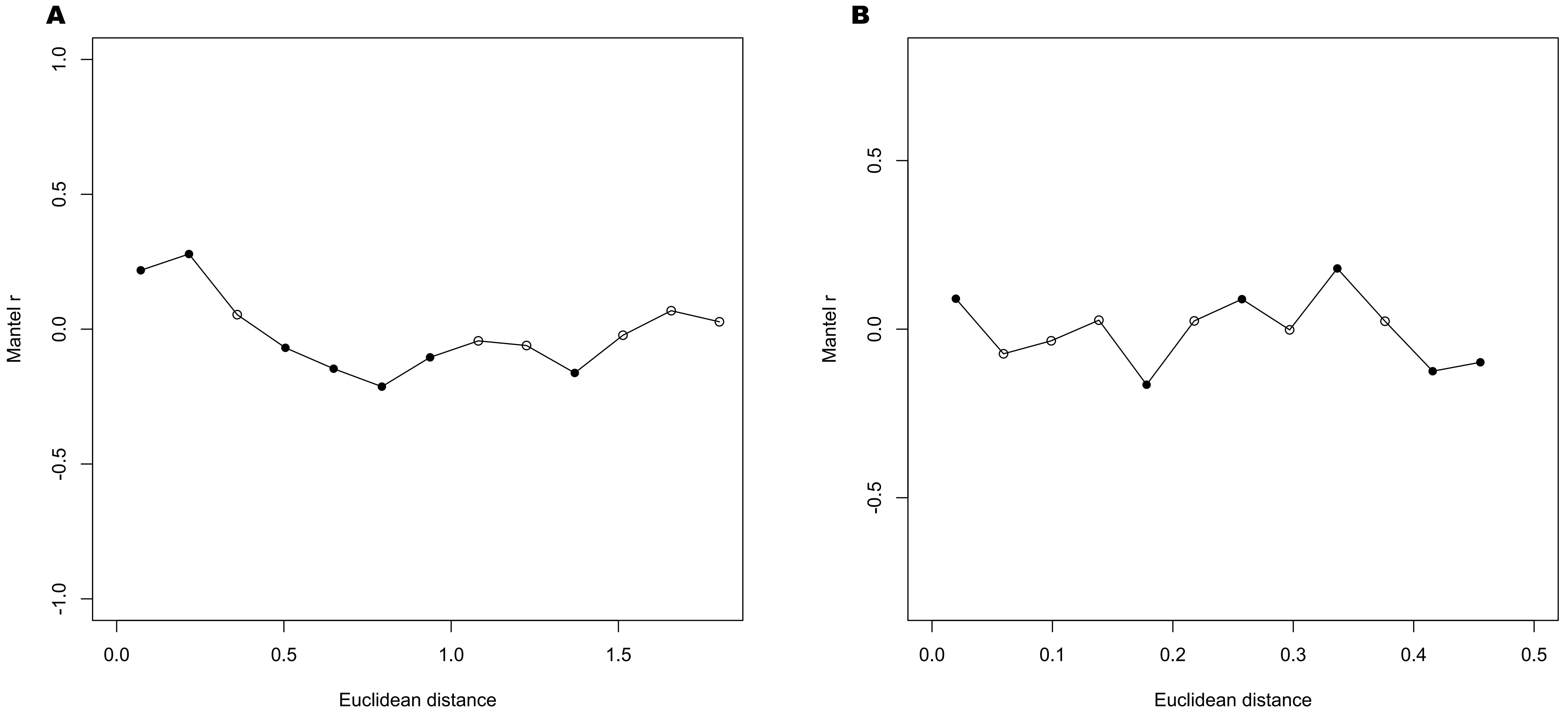

Supplement: Figure S1 — Mantel test for a) HO-FULL and b) HO-ANA showing variation in r in different distance classes. (PDF) [file pone.0071573.s001.pdf]
