# Supplementary material for: Flying between Sky Islands: The Effect of Naturally Fragmented Habitat on Butterfly Population Structure
Source: PLoS One. 2013 Aug 1;8(8):e71573. doi: 10.1371/journal.pone.0071573 (PMC3731288; doi:10.1371/journal.pone.0071573)

Figure S2. Eigenvalues and screeplots for the sPCA analyses

a) sPCA plots for *Mycalesis patnia*.

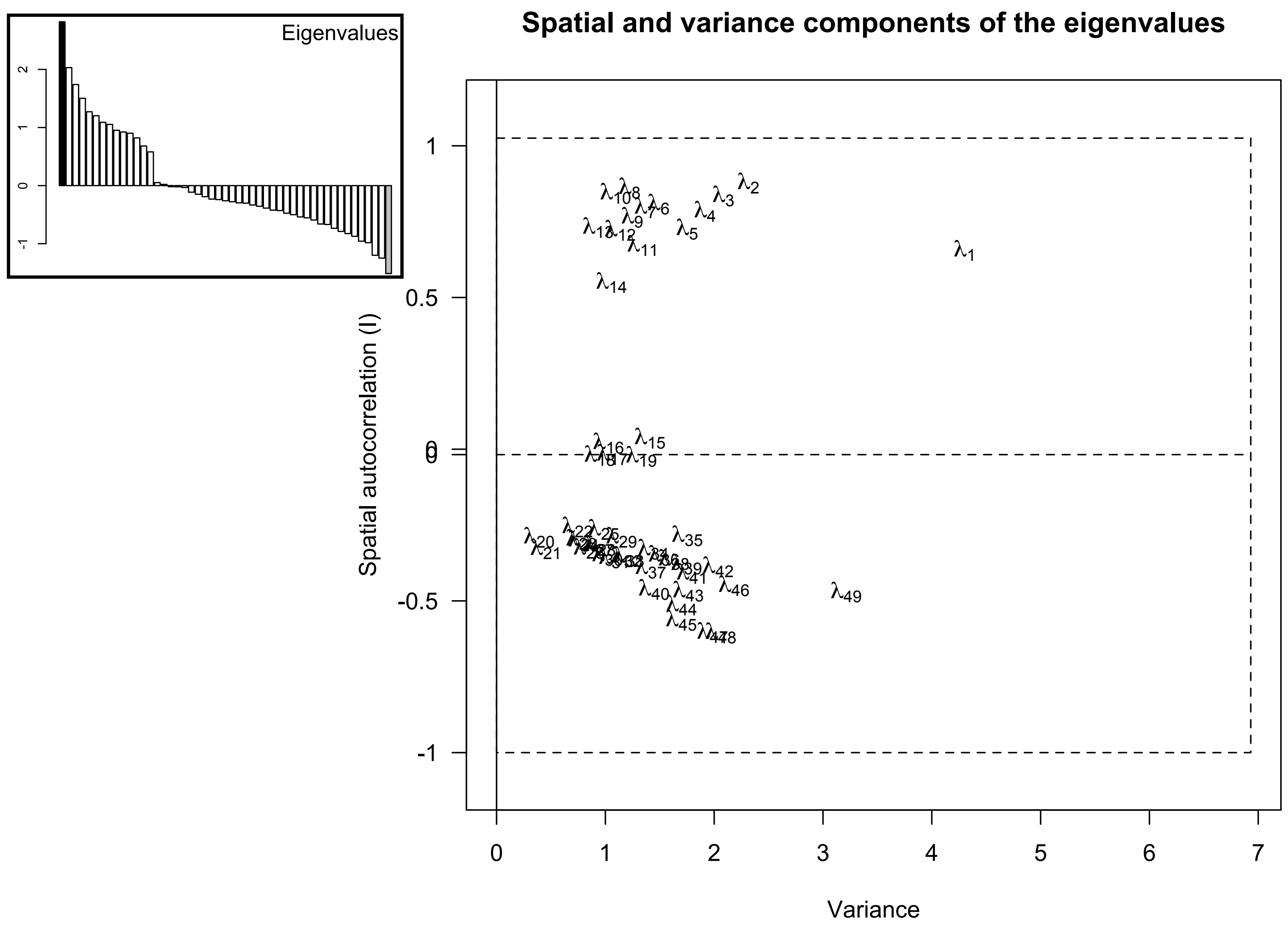

b) sPCA plots for *Heteropsis oculus*.

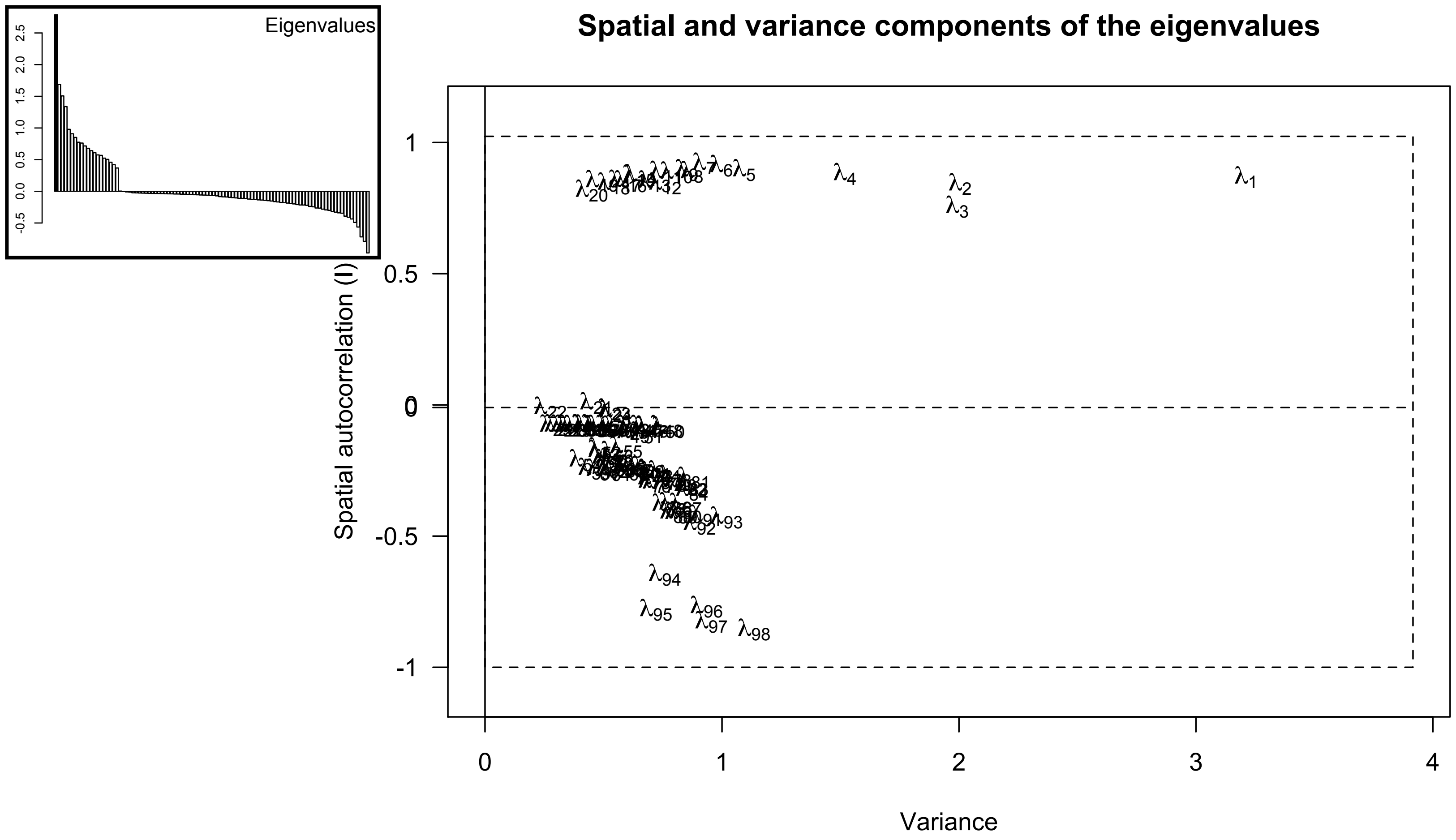

c) sPCA plots for the Anamalai population of *Heteropsis oculus*.

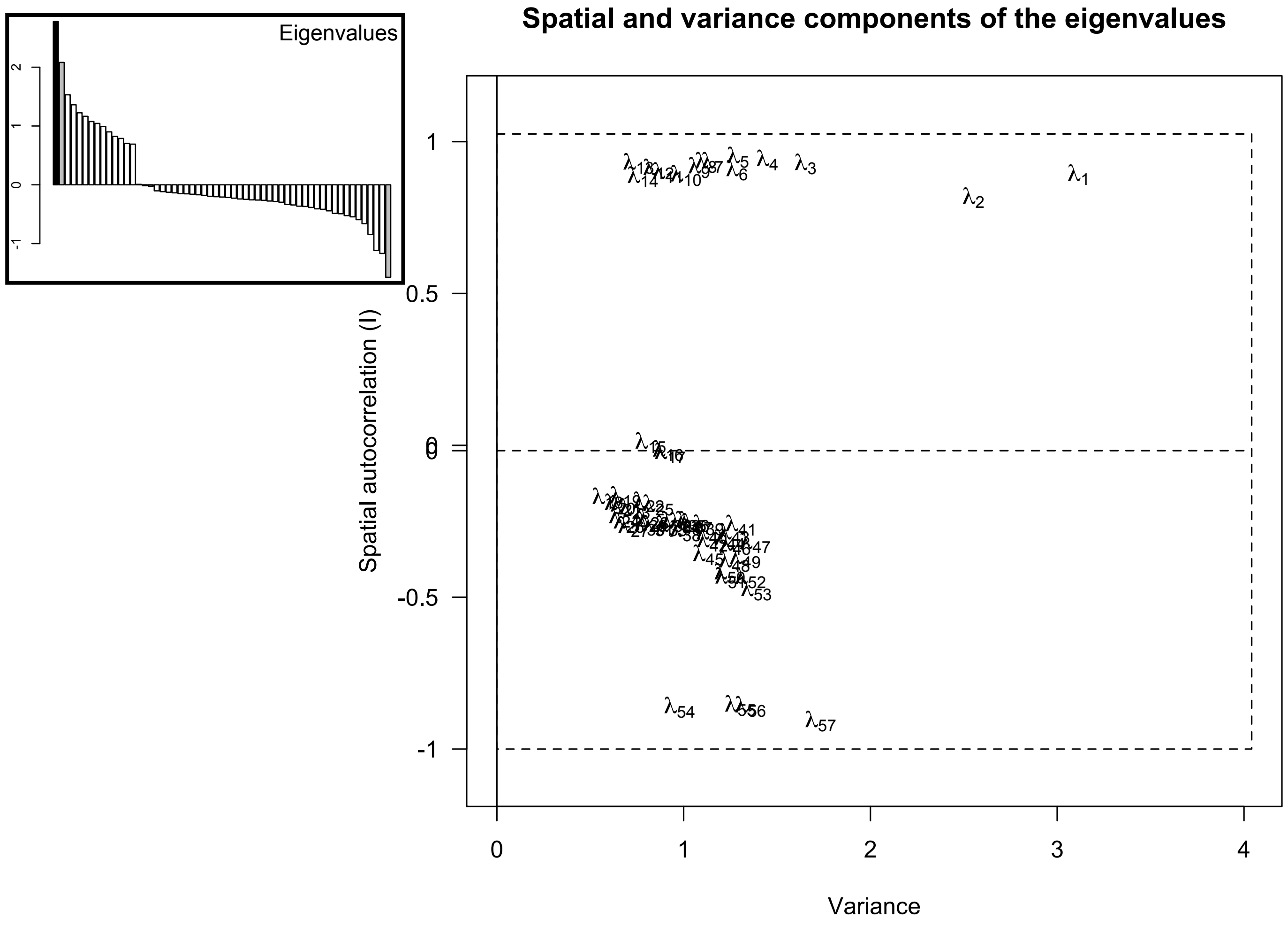

Supplement: Figure S2 — Eigenvalues and screeplots for the sPCA analyses. (PDF) [file pone.0071573.s002.pdf]
