# Supplementary material for: Flying between Sky Islands: The Effect of Naturally Fragmented Habitat on Butterfly Population Structure
Source: PLoS One. 2013 Aug 1;8(8):e71573. doi: 10.1371/journal.pone.0071573 (PMC3731288; doi:10.1371/journal.pone.0071573)
Supplement: Table S1 — Sampling locations for both species. (PDF) [file pone.0071573.s003.pdf]

Table S1. Sampling locations for both species

a) ***Heteropsis oculus***

Please affix CES in front of the Sample Codes.

S.No.	Sample code	Sampling location	Latitude	Longitude	Population
1	09/694	Pandimatta, Shendurney	8.8727	77.1634	AGA
2	09/695	Pandimatta, Shendurney	8.8727	77.1634	AGA
3	09/696	Pandimatta, Shendurney	8.8727	77.1634	AGA
4	09/697	Pandimatta, Shendurney	8.8727	77.1634	AGA
5	10/512	Chemmunji hut	8.7507	77.1744	AGA
6	10/513	Chemmunji	8.7507	77.1744	AGA
7	10/514	Chemmunji	8.7507	77.1744	AGA
8	10/765	Pambadam shola	10.135	77.2628	ANA
9	10/789	Pambadam shola	10.125	77.2576	ANA
10	10/790	Pambadam shola	10.125	77.2576	ANA
11	10/791	Mannavan shola	10.177	77.1902	ANA
12	10/792	Mannavan shola	10.177	77.1902	ANA
13	10/793	Mannavan shola	10.177	77.1902	ANA
14	11/501	Akkamalai	10.332	77.022	ANA
15	11/502	Akkamalai	10.332	77.022	ANA
16	11/503	Akkamalai	10.332	77.022	ANA
17	11/504	Akkamalai	10.332	77.022	ANA
18	11/513	Mannavan shola	10.177	77.1902	ANA
19	11/514	Mannavan shola	10.177	77.1902	ANA
20	11/515	Mannavan shola	10.177	77.1902	ANA
21	11/516	Mannavan shola	10.177	77.1902	ANA
22	11/517	Rajmala, Eravikulam	10.168	77.023	ANA
23	11/518	Rajmala, Eravikulam	10.168	77.023	ANA
24	11/519	Rajmala, Eravikulam	10.168	77.023	ANA
25	11/520	Rajmala, Eravikulam	10.168	77.023	ANA

26	11/522	Rajmala, Eravikulam	10.136	77.034	ANA
27	11/523	Rajmala, Eravikulam	10.136	77.034	ANA
28	11/524	Vaguvaraiyar, Eravikulam	10.185	77.1411	ANA
29	11/525	Vaguvaraiyar, Eravikulam	10.185	77.1411	ANA
30	11/526	Vaguvaraiyar, Eravikulam	10.185	77.1411	ANA
31	11/527	Vaguvaraiyar, Eravikulam	10.185	77.1411	ANA
32	11/528	Vaguvaraiyar, Eravikulam	10.185	77.1411	ANA
33	11/529	Vaguvaraiyar, Eravikulam	10.172	77.1221	ANA
34	11/530	Vaguvaraiyar, Eravikulam	10.172	77.1221	ANA
35	11/531	Vaguvaraiyar, Eravikulam	10.172	77.1221	ANA
36	11/532	Vaguvaraiyar, Eravikulam	10.172	77.1221	ANA
37	11/533	Vaguvaraiyar, Eravikulam	10.172	77.1221	ANA
38	11/534	Pambadam shola	10.131	77.2615	ANA
39	11/535	Pambadam shola	10.131	77.2615	ANA
40	11/536	Pambadam shola	10.131	77.2615	ANA
41	11/537	Pambadam shola	10.142	77.2549	ANA
42	11/538	Pambadam	10.142	77.2549	ANA
43	11/539	Pambadam shola	10.142	77.2549	ANA
44	11/543	Mathikettan shola	9.9713	77.2328	ANA
45	11/544	Mathikettan shola	9.9723	77.2425	ANA
46	11/545	Mathikettan shola	9.9723	77.2425	ANA
47	11/546	Mathikettan shola	9.9723	77.2425	ANA
48	11/547	Mathikettan shola	9.9741	77.2470	ANA
49	11/548	Mathikettan shola	9.9741	77.2470	ANA
50	11/549	Mathikettan shola	9.9755	77.2411	ANA
51	11/550	Mathikettan shola	9.9755	77.2411	ANA
52	11/551	Mathikettan shola	9.9755	77.2411	ANA
53	11/552	Mathikettan shola	9.9755	77.2411	ANA
54	11/553	Mathikettan shola	9.9755	77.2411	ANA
55	11/568	Pandimatta, Shendurney	8.8727	77.1634	AGA
56	11/575	Pandimatta, Shendurney	8.8727	77.1634	AGA

57	11/576	Pandimatta, Shendurney	8.8727	77.1634	AGA
58	11/577	Pandimatta, Shendurney	8.8727	77.1634	AGA
59	11/578	Pandimatta, Shendurney	8.8727	77.1634	AGA
60	11/579	Pandimatta, Shendurney	8.8727	77.1634	AGA
61	11/584	Pandimatta, Shendurney	8.8727	77.1634	AGA
62	11/585	Pandimatta, Shendurney	8.8727	77.1634	AGA
63	11/586	Pandimatta, Shendurney	8.8727	77.1634	AGA
64	11/589	Pandimatta, Shendurney	8.8727	77.1634	AGA
65	11/598	Poomparai, Kodaikanal	10.259	77.3797	ANA
66	11/599	Bombay shola, Kodaikanal	10.229	77.486	ANA
67	11/600	Bombay shola, Kodaikanal	10.229	77.486	ANA
68	11/601	Berijam, Kodaikanal	10.207	77.4418	ANA
69	11/602	Berijam, Kodaikanal	10.207	77.4418	ANA
70	11/603	Berijam, Kodaikanal	10.207	77.4418	ANA
71	11/604	Berijam, Kodaikanal	10.207	77.4418	ANA
72	11/605	Berijam, Kodaikanal	10.207	77.4418	ANA
73	11/606	Berijam lake, Kodaikanal	10.182	77.3935	ANA
74	11/607	Berijam lake, Kodaikanal	10.182	77.3935	ANA
75	11/608	Berijam lake, Kodaikanal	10.182	77.3935	ANA
76	11/609	Vattaparai, High Wavies	9.5845	77.3317	MEGH
77	11/610	Vattaparai, High Wavies	9.5845	77.3317	MEGH
78	11/611	Melmanalar, High Wavies	9.6011	77.3475	MEGH
79	11/612	Melmanalar, High Wavies	9.6011	77.3475	MEGH
80	11/613	Melmanalar, High Wavies	9.6011	77.3475	MEGH
81	11/614	Melmanalar, High Wavies	9.6011	77.3475	MEGH
82	11/615	Melmanalar, High Wavies	9.6011	77.3475	MEGH
83	11/616	Melmanalar, High Wavies	9.6011	77.3475	MEGH
84	11/617	Melmanalar, High Wavies	9.6011	77.3475	MEGH
85	11/618	Melmanalar, High Wavies	9.6011	77.3475	MEGH
86	11/619	Melmanalar, High Wavies	9.6011	77.3475	MEGH
87	11/620	Melmanalar, High Wavies	9.6011	77.3475	MEGH

88	11/621	Melmanalar, High Wavies	9.6011	77.3475	MEGH
89	11/622	Melmanalar, High Wavies	9.6011	77.3475	MEGH
90	11/623	Melmanalar, High Wavies	9.6011	77.3475	MEGH
91	11/624	Melmanalar, High Wavies	9.6011	77.3475	MEGH
92	11/641	Pandian estate, Vellimalai	9.5333	77.3701	MEGH
93	11/642	Pandian estate, Vellimalai	9.5333	77.3701	MEGH
94	11/643	Pandian estate, Vellimalai	9.5333	77.3701	MEGH
95	11/644	Pandian estate, Vellimalai	9.5333	77.3701	MEGH
96	11/654	KMTR	8.5295	77.3298	AGA
97	11/655	KMTR	8.4927	77.3812	AGA
98	11/656	KMTR	8.4927	77.3812	AGA
99	11/657	KMTR	8.4927	77.3812	AGA

Here, KMTR: Kalakkad Mundanthurai Tiger Reserve.

Populations: AGA – Agastyamalai and surrounding sky islands, including KMTR, Chemmunji and Pandimatta, forming the southernmost sky island complex; ANA – Anamalais, including sholas in Kodaikanal, Munnar and Valparai; MEGH – Meghamalais, including Vellimalai and High Wavies estate.

b) ***Mycalesis patnia***

Please affix CES in front of the Sample Codes.

**S.No.**	**Sample code**	**Sampling location**	**Latitude**	**Longitude**	**Population**
1	08/521	Vazhani	10.303	76.593	ANA
2	08/624	Karikan (Coorg)	12.29268	75.603272	WAC
3	08/691	Kudremukh	12.29268	75.603272	WAC
4	08/738	Nelliampathy	10.53405	76.65161	ANA
5	09/666	Vazhachal	10.30344	76.59327	ANA

6	09/687	Rockwood	8.87392	77.1028	AGA
7	09/693	Pandimatta	8.87275	77.1634	AGA
8	09/703	Kattalapara	8.91898	77.09629	AGA
9	09/708	Pampoori (Thattekad)	10.11827	76.71546	ANA
10	09/748	Pandimatta	8.87275	77.1634	AGA
11	09/749	Pandimatta	8.87275	77.1634	AGA
12	09/750	Pandimatta	8.87275	77.1634	AGA
13	09/758	Pasikala (Shendurney)	8.87392	77.1028	AGA
14	09/759	Pasikala (Shendurney)	8.87392	77.1028	AGA
15	09/771	Anshi	15.01697	74.38997	NKAR
16	09/774	Kulgi	15.16811	74.6343	NKAR
17	09/789	Thattekad	10.11827	76.71546	ANA
18	09/792	Thattekad	10.11827	76.71546	ANA
19	09/800	Thattekad	10.11827	76.71546	ANA
20	10/702	Kulgi	15.16811	74.6343	NKAR
21	10/703	Kulgi	15.16811	74.6343	NKAR
22	10/704	Agumbe	13.5134	75.082	NKAR
23	10/706	Agumbe	13.5134	75.082	NKAR
24	10/707	Agumbe	13.5134	75.082	NKAR
25	11/505	Karian shola, Perambikulam	10.444	76.866	ANA
26	11/506	Karian shola, Perambikulam	10.444	76.866	ANA
27	11/507	Salim Ali point, Perambikulam	10.352	76.767	ANA
28	11/508	Salim Ali point, Perambikulam	10.352	76.767	ANA
29	11/509	Salim Ali point, Perambikulam	10.352	76.767	ANA
30	11/540	Mathikettan shola	10.029	77.129	ANA
31	11/541	Mathikettan shola	10.029	77.129	ANA

32	11/542	Mathikettan shola	10.029	77.129	ANA
33	11/563	Mundakkai, Wayanad	11.562	76.133	WAC
34	11/564	Mundakkai, Wayanad	11.562	76.133	WAC
35	11/565	Lady Smith, Wayanad	11.66	75.933	WAC
36	11/588	Achenkovil	9.111	77.156	AGA
37	11/596	Gudrikkal	9.386	77.15	MEGH
38	11/597	Gudrikkal	9.386	77.15	MEGH
39	11/626	Varushanad hills, Vellimalai	9.5685	77.4022	MEGH
40	11/627	Varushanad hills, Vellimalai	9.5685	77.4022	MEGH
41	11/628	Varushanad hills, Vellimalai	9.5685	77.4022	MEGH
42	11/629	Varushanad hills, Vellimalai	9.5685	77.4022	MEGH
43	11/630	Varushanad hills, Vellimalai	9.5685	77.4022	MEGH
44	11/631	Varushanad hills, Vellimalai	9.5685	77.4022	MEGH
45	11/649	Kuthiraivetty, KMTR	8.5944	77.359	AGA
46	11/650	Kuthiraivetty, KMTR	8.5944	77.359	AGA
47	11/651	Kuthiraivetty, KMTR	8.5944	77.359	AGA
48	11/652	Kuthiraivetty, KMTR	8.5944	77.359	AGA
49	11/653	Kuthiraivetty, KMTR	8.5944	77.359	AGA
50	11/669	Achenkovil	9.111	77.156	AGA
51	11/670	Gudrikkal	9.386	77.15	MEGH
52	11/671	Gudrikkal	9.386	77.15	MEGH
53	11/672	Moozhiyar	9.31774	77.08242	MEGH
54	11/673	Moozhiyar	9.31774	77.08242	MEGH
55	11/674	Moozhiyar	9.31774	77.08242	MEGH
56	11/675	Moozhiyar	9.31774	77.08242	MEGH

57	11/676	Moozhiyar	9.31774	77.08242	MEGH

Here, KMTR: Kalakkad Mundanthurai Tiger Reserve.

Populations: AGA, ANA and MEGH – lower elevation areas around Agastyamalai, Anamalai and Meghamalai sky island complexes; WAC – Wayanad and Coorg; NKR – North Karnataka.
