# Supplementary material for: Flying between Sky Islands: The Effect of Naturally Fragmented Habitat on Butterfly Population Structure
Source: PLoS One. 2013 Aug 1;8(8):e71573. doi: 10.1371/journal.pone.0071573 (PMC3731288; doi:10.1371/journal.pone.0071573)
Supplement: Table S2 — Full results of the spatial autocorrelation analysis. (PDF) [file pone.0071573.s004.pdf]

Table S2. Full results of the spatial autocorrelation analysis.

Here, U and L denote the upper and lower limits of *r* as per the permutation tests. The observed *r* values for both species that fall outside the confidence limits of the permutation tests, and are marked as significant (**§**). *P* is the probability of existence of spatial structure (both positive and negative), according to one tailed tests, and is marked with a * if significant.

a) The MP dataset

**Distance Class (km, mid point)**	**50**	**150**	**250**	**350**	**450**	**550**	**650**	**750**
r	0.004	-0.006	-0.005	0.016	0.004	0.003	-0.024 §	0.005
**U**	0.006	0.009	0.017	0.019	0.031	0.021	0.022	0.024
**L**	-0.005	-0.010	-0.016	-0.020	-0.024	-0.017	-0.022	-0.021
***P* (positive spatial autocorrelation)**	0.099	0.903	0.721	0.054	0.349	0.381	0.983	0.342
***P* (negative spatial autocorrelation)**	0.902	0.098	0.280	0.947	0.652	0.620	0.018*	0.659

b) The HO dataset

**Distance Class (km, mid point)**	**25**	**75**	**125**	**175**	**225**
r	0.003	-0.007	0.008 §	-0.010	0.031
**U**	0.003	0.002	0.004	0.004	0.033
**L**	-0.002	-0.003	-0.004	-0.005	-0.031
***P* (positive spatial autocorrelation)**	0.003**	1.000	< 0.0001***	1.000	0.029*

c) The HO-ANA dataset

**Distance Class (km, mid point)**	**5**	**15**	**25**	**35**	**45**	**55**
r	0.005	0.002	-0.001	0.004	-0.021 **§**	-0.035 **§**
**U**	0.006	0.005	0.004	0.007	0.009	0.026
**L**	-0.005	-0.006	-0.005	-0.007	-0.010	-0.026
***P* (positive spatial autocorrelation)**	0.043	0.233	0.636	0.107	1.00	0.994
***P* (negative spatial autocorrelation)**	0.958	0.767	0.365	0.893	< 0.0001**	0.006*
