# Supplementary material for: Flying between Sky Islands: The Effect of Naturally Fragmented Habitat on Butterfly Population Structure
Source: PLoS One. 2013 Aug 1;8(8):e71573. doi: 10.1371/journal.pone.0071573 (PMC3731288; doi:10.1371/journal.pone.0071573)
Supplement: Table S3 — Band-based FST values between populations in all datasets. (PDF) [file pone.0071573.s005.pdf]

Table S3. Band-based F_{ST} values between populations of the a) MP b) HO and b) HO-ANA datasets. Significant F_{ST} values are in bold.

a) MP dataset

Populations	AGA	ANA	MEGH	NKAR	WAC
AGA	-				
ANA	**0.03306**	-			
MEGH	0.02151	0.00629	-		
NKAR	0.04273	0.00248	-0.00805	-	
WAC	0.02344	0.01322	-0.01558	0.0093	-
**Overall F_{ST} = 0.015, P = 0.03**					

b) HO dataset

Populations	AGA	ANA	MEGH
AGA	-		
ANA	**0.02995**	-	
MEGH	**0.04664**	**0.05664**	-
**Overall F_{ST} = 0.043, P < 0.0001**			

c) HO-ANA dataset

Populations	AKK	KODI	MANN	MATH	PAM	RAJ	VAG
AKK	-						
KODI	**0.083**	-					
MANN	-0.008	**0.051**	-				
MATH	0.003	**0.096**	**0.020**	-			
PAM	-0.019	**0.058**	-0.025	0.006	-		
RAJ	-0.012	**0.078**	0.012	0.019	-0.002	-	
VAG	-0.001	**0.093**	-0.001	0.002	-0.012	-0.013	-
**Overall F_{ST} = 0.029, P < 0.0001**							
